# Microbiota–Liver Diseases Interactions

**DOI:** 10.3390/ijms24043883

**Published:** 2023-02-15

**Authors:** Rosanna Capparelli, Paola Cuomo, Antonio Gentile, Domenico Iannelli

**Affiliations:** Department of Agriculture Sciences, University of Naples Federico II, 80055 Naples, Italy

**Keywords:** gut–microbiota, pathobionts, liver diseases

## Abstract

Gut microbiota regulates essential processes of host metabolism and physiology: synthesis of vitamins, digestion of foods non-digestible by the host (such as fibers), and—most important—protects the digestive tract from pathogens. In this study, we focus on the CRISPR/Cas9 technology, which is extensively used to correct multiple diseases, including liver diseases. Then, we discuss the non-alcoholic fatty liver disease (NAFLD), affecting more than 25% of the global population; colorectal cancer (CRC) is second in mortality. We give space to rarely discussed topics, such as pathobionts and multiple mutations. Pathobionts help to understand the origin and complexity of the microbiota. Since several types of cancers have as target the gut, it is vital extending the research of multiple mutations to the type of cancers affecting the gut–liver axis.

## 1. Introduction

The gut microbiota of healthy individuals is similar to abundance and bacterial species. The human gut contains bacterial species useful to the host, but also pathobionts. The former species digest foods not digestible by humans (such as fibers), synthesize vitamins, and—importantly—protect the digestive tract of the host from pathogens [1]. Pathobionts are opportunistic microorganisms that expand in case of perturbation in the useful fraction of the microbiota. Pathobionts prevalence leads to dysbiosis: a disproportion in the gut microbiota due to earning or loss of community members or changes in their abundance [2]. Dysbiosis causes microbial alteration in the gut microbiota, increasing the number of Gram-negative bacteria and producing lipopolysaccharide (LPS). As a pathogen-associated molecular pattern (PAMP), LPS initiates the host inflammatory response through the activation of the TLR4. Accumulation of LPS, due to gut permeability and translocation of bacterial components, is a relevant health risk contributing to the development of several diseases, including metabolic dysfunction-associated fatty liver disease (MAFLD), type 2 Diabetes, kidney disease, obesity, and inflammation [3,4].

The liver plays a central role in carbohydrate, lipid, protein metabolism, and nutrient catabolism, converting them into substances essential for the body [5]. In addition, the liver detoxifies natural metabolites, such as ammonia and bilirubin. Bile acids (BAs), synthesized from cholesterol in the liver, are indispensable for cholesterol metabolism and lipid digestion [6]. BAs are secreted in the intestine during food digestion [7], then are reabsorbed in the ileum and conveyed back to the liver through the portal vein. BAs promote the absorption of dietary fats, cholesterol, and fat-soluble vitamins [7]. As signaling molecules, BAs—activating the farnesoid X receptor (FXR) and the binding of the G-protein-coupled bile acid receptor 1 [8,9,10]—regulate glucose and lipid metabolism. BAs also interact with the microbiota; specifically, they supervise the intestinal mucosal integrity and synthesis of antibacterial peptides [11]. Further, the binding of BAs to FXR induces the synthesis of antimicrobial peptides (such as angiotensin 1) that inhibit microbiota overgrowth by increasing the intestinal epithelial cell capability to prevent bacterial uptake [11]. In turn, the microbiota can influence the size and composition of the BAs population, converting primary to secondary BAs [12,13]. This change in the composition of the circulating BAs alters lipid and glucose metabolism and predisposes individuals to non-alcoholic fatty liver disease (NAFLD). Thus, both the altered equilibrium of microbiota and BAs may lead to liver diseases [12,13]. Not surprisingly, the multiple roles of the liver involve it in many inherited metabolic diseases (IMDs). Alteration of metabolic pathways regulated by the liver causes accumulation of toxic by-products and hence liver damage. At present, IMDs are more than 400 [14]. The majority of these diseases are autosomal recessive [caused by a single allele mutation located in one of the autosomal (not sex) chromosomes]; their frequency is approximately one in 800 newborns. Liver transplantation solves many cases of IMDs. This result indicates that replacing mutated alleles solves part of IMD cases [15]. However, the scarcity of donors and the necessity of immunosuppressing the transplant recipient limit transplantation to very few patients [16]. This review focuses on the role of gut microbiota in liver diseases. Specifically: (1) Non-alcoholic fatty liver disease: the most frequent cause of liver diseases; (2) colorectal cancer: second in mortality; (3) multiple mutations: cancer often selects the gut as a target; therefore, is urgently studying these somatic cis-regulator mutants; (4) an attempt to rationalize the multiple and often opposite interactions between gut microbiota, pathogens, and host; an issue stressful for students.

## 2. CRISPR/Cas9: The Most Outstanding Results

**Figure 1:**
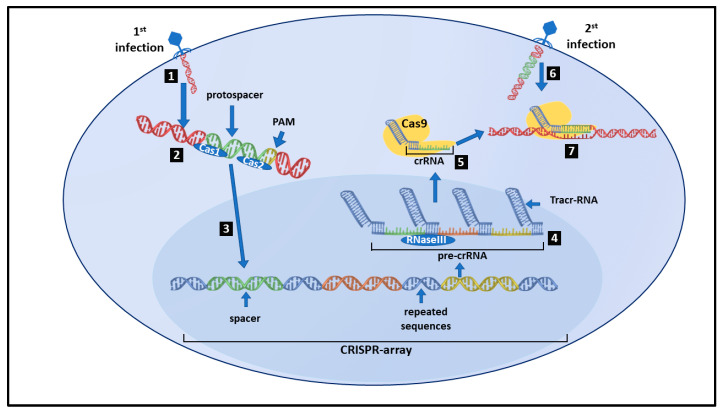
CRISPR/Cas system in bacteria. When a bacteriophage infects a bacterium for the first time (1), its DNA is scanned by Caspase proteins 1 and 2, which recognize specific viral sequences located near the protospacer adjacent motif (PAM) (2). Once a PAM is recognized, DNA is cleaved upstream the PAM sequence and protospacer is integrated into the CRISPR-array, which includes hexogen DNA (spacers) intercalated between bacterial DNA palindromic repeated sequences (3). The CRISPR-array transcribed by bacterium (pre-crRNA) complements with trans-activating RNA (tracr-RNA) encoded in proximity of the Cas genes (4). The RNaseIII recognizes this complex and cleaves it into smaller RNAs named CRISPR-RNA (crRNA). The crRNA is bound by Caspase9 (Cas9) forming the ribonucleoprotein complex named CRISPR/Cas9 I (5). Once the same bacteriophage tries to infect the host cell for the second time (6), the CRISPR/Cas9 complex recognizes PAM and cleaves the viral DNA (7).

Recently, several therapies able to edit mutated genes have been developed [17]. In 2021, a patient with methylmalonic academia (MMA) (a rare liver disease) underwent treatment with nanoparticle-carrying methylmalonic mutase (MUT) mRNA to test the security and tolerance of the therapy. Among the most successful approaches, the ones relying on Clustered Regularly Interspaced Short Palindromic Repeats (CRISPR) technology seem to be very promising. CRISPR is part of the immunity system of archaea against bacteriophages, their main competitors [18]. Due to its capability to carry out specific DNA cleavage, this technique allows identifying which gene(s) causes the diseases (Figure 1). Once infected by a bacteriophage, the Cas proteins (Cas1 and Cas2) inspect the viral DNA to recognize the special sequence of viral DNA named protospacer, which is cleaved by the Cas proteins and integrated into the bacterial DNA. The DNA segment containing the viral protospacer is cleaved into small RNA segments [CRISPR-RNA (crRNAs)] and bound to the Cas9 protein forming the ribonucleoprotein complex (CRISPR/Cas9). If the crRNA sequence is complementary to viral DNA, the latter is cut and inactivated [19]. In addition, CRISPR is extensively used to knockout target genes in different cell types and organisms, thus representing a precise approach to studying gene function [20].

This approach was achieved by Tian et al. in order to disclose the role of *UGT1A9*, a gene encoding a UDP-glucuronosyltransferase enzyme, in bisphenol-induced NAFLD [21]. They observed limited liver injuries in *UGT1A^−/−^* mice when exposed to bisphenol, compared to wild-type mice. This result displayed the critical role of *UGT1A9* in bisphenol-mediated mitochondrial dyshomeostasis and NAFLD pathogenesis [21]. Recent advances made CRISPR technology a powerful tool able to target multiple genes simultaneously and study their combinatory effects [22,23]. *PNPLA3*, *TM6SF2*, and *MBOAT7* genes are involved in the pathogenesis and progression of NAFLD [24]. In detail, variants *rs738409* in *PNPLA3*, *rs641738* in *MBOAT7*, and *rs58542926* in *TM6SF2* are associated with increased triglyceride production, accumulation of very low-density lipoprotein (VLDL) and triglycerides and increased risk of developing cirrhosis, respectively [25]. In order to assess the association between the above SNPs and the severity of liver diseases, Longo et al. silenced HepG2 cells for *rs738409 PNPLA3*, *rs641738 MBOAT7*, and *rs58542926 TM6SF2* using the CRISPR/Cas9 technology [26]. Results showed that *MBOAT7^−/−^* and *TM6SF2^−/−^* models inhibited the release of both Apolipoprotein B and TAG-rich lipoproteins and reduced the risk and severity of fatty liver diseases [26].

**Figure 2:**
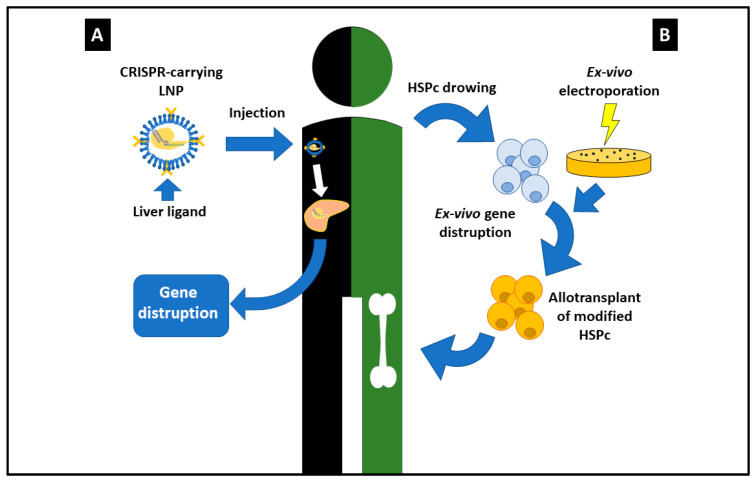
In vivo CRISPR applications. The (**A**) part of the figure represents a scheme of CRISPR/Cas9 complex incapsulated into Lipid Nanoparticles (LNPs). The LNPs are coated with apolipoprotein E (apoE) which will lead the particle into the liver. After intravenous administration, the complex reaches the liver and is taken by the hepatocytes through the interaction with apolipoprotein E. Then CRISPR/Cas9 is released and leads to double strands breaks in the gene which will lose function. In the right side of figure (**B**) is depicted the method for the knockout of the *BCLA11A* promoter in β-thalassemia. After isolation of hematopoietic stem and progenitor cells (HSPc) from the patient, CRISPR/Cas9 complex has been inserted in these cells ex vivo through electroporation. The cells in which the gene loses its function are reimplanted into the patient. These cells will colonize the bone-narrow without producing *BCLA11A* which causes the disease.

Independent research also reported the effectiveness of CRISPR/Cas genome editing technology in treating human diseases [27]. The treatment with CRISPR-Cas editing technology of patients suffering from transthyretin amyloidosis (ATTR) yielded very encouraging results [28]. The encapsulation of the CRISPR/Cas9 complex into lipid nanoparticles (LNP) corrected the transthyretin amyloid (ATTR) disease due to the over-expression of the transthyretin (*TTR*) gene. The LNP-encapsulated CRISPR was therefore inoculated intravenously and adsorbed by host cells, where the complex cuts away the *TTR* gene (Figure 2A). The large volume of blood flowing in the liver favors the accumulation of the gene therapy particles directly in the liver hepatocytes through their fenestrated endothelium more rapidly than in organs with a continuous endothelium. In addition, the very slow overturn of hepatocytes provides limited dilution of gene therapy. Patients with one dose of 0.3 mg/kg of CRISPR-Lipid nanoparticles showed a reduction of 87% in TTR serum protein.

Remarkable results were also obtained with β-thalassemia, a disease caused by over-expression of the *BCLA11A* gene [29]. Hematopoietic stem and progenitor cells (HSPc) were corrected ex-vivo by knocking out the *BCLA11A* promoter and then reimplanted in the patients (Figure 2B). Notwithstanding adverse effects, such as neutropenia, abdominal pain, and pneumonia, both patients have had persistent hemoglobin expression for over one year following administration without the necessity of a blood transfusion. In conclusion, much progress has been gained in treating IMDs since the dietary control of phenylketonuria in 1950.

Along with such outstanding techniques, it is also justified to mention the contribution of mice and other rodents to the study of liver diseases. Born by caesarian section and grown in a sterile condition, these mice (germ-free) have an unfinished immune system and a permeable gut barrier. Conventional mice with a permeable gut barrier are prone to liver diseases. Instead, germ-free mice with similar permeability are resistant [30]. A plausible explanation of this finding is that germ-free mice—protected from the presence of bacteria—cannot translocate LPS or other toxic bacterial components. This case well explains how microbiota can protect against or cause diseases.

## 3. Non-Alcoholic Fatty Liver Disease (NAFLD)

Non-alcoholic fatty liver disease (NAFLD) is a pathological condition affecting approximately 25% of the global population [31]. It is the most common cause of chronic liver disorders and can easily evolve into cirrhosis and liver cancer [32]. NAFLD is characterized by the accumulation of triglycerides in liver cells. Therefore, it is not surprising that diet plays a critical role in the pathogenesis of NAFLD, and non-alcoholic steatohepatitis (NASH) is a more severe form of NAFLD. In addition to genetic and metabolic factors, altered gut microbiota also contributes to NAFLD progression [33].

Significant gut microbiota alterations characterize NAFLD patients. In vivo studies revealed an abundance of *Firmicutes* in NAFLD patients. Conversely, *Firmicutes* are decreased in children with NASH, while levels of *Bacteroides*, *Proteobacteria*, and *Enterobacteria* are increased [34].

**Figure 3:**
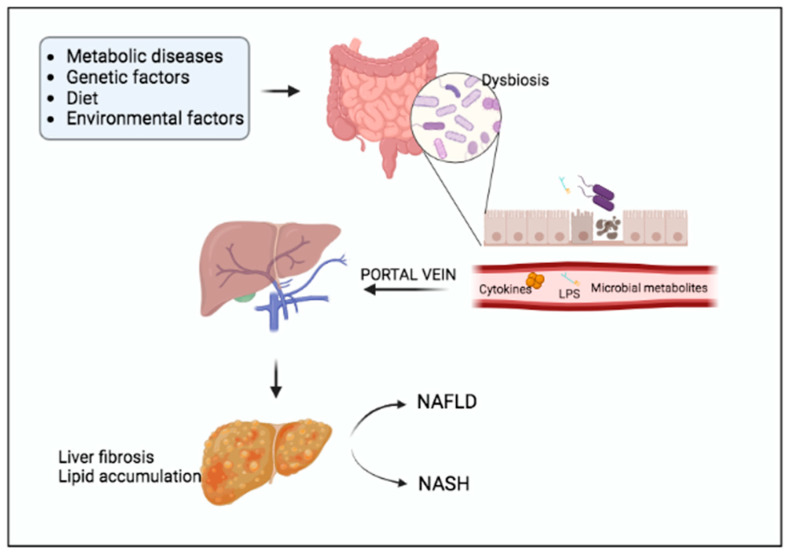
The gut microbiota dysbiosis induces NAFLD progression. The gut microbiota alterations associated with metabolic, genetic or environmental factors play key roles in liver disorders. Dysbiosis increases gut permeability, favors LPS translocation and promotes pro-inflammatory cytokines production, thus participating to liver fibrosis and NAFLD occurrence.

Commonly, dysbiosis alters gut permeability, promoting gut microbiota translocation and liver inflammation as a consequence of lipopolysaccharide (LPS) accumulation (Figure 3) [3].

A clear demonstration of the role of LPS is provided by Csak et al. [35]. The authors show that mice—knockout for the LPS receptor genes (*TLR4* and/or *MD-2*) and fed with a methionine–choline-deficient (MCD) diet—do not develop liver fibrosis. This is congruent with the property of MCD to favor fat liver accumulation and NASH progression. Interestingly, the *Lactobacillus casei* inhibits liver inflammation and protects against NASH following an MCD diet [36]. Fecal microbiota transplantation (FMT) represents a valid approach to reducing the NAFLD clinical manifestations and restoring homeostasis [37]. Butyrate is a short-chain fatty acid (SCFA) produced by *Firmicutes* and *Bacteroides* during the fermentation of dietary fibers [38]. This microbial metabolite contributes to the host’s metabolic health by reducing the gut permeability and, consequently, the concentration of LPS in the serum [39]. Fecal transplantation from C57BL/6 mice increases the intestinal concentration of butyrate and protects against NASH and NAFLD [39]. The role of gut microbiota in NAFLD and NASH progression is further confirmed when the experiments are carried out in germ-free mice rather than conventional biochemical approaches. Recent studies demonstrate that germ-free C57BL/6J mice are protected from fatty liver accumulation when consuming a high-carbohydrate diet, high-fat diet, high-fructose diet, or Western diet (41% carbohydrates and 41% fat) [40,41,42]. Similarly, germ-free Fischer rats exhibit less fat in the liver when receiving a choline-deficient, low-cystine, or low-cholesterol diet. On the contrary, they develop severe hepatic steatosis after a choline-deficient, high-cystine, high-cholesterol diet [43]. These results can plausibly be attributed to the absence in the microbiota of bacterial species capable of metabolizing cystine and cholesterol or, alternatively, to the presence of beneficial metabolites.

Further, the protective effect described in germ-free C57BL/6J mice can be explained assuming the absence of gut microbiota and, thus, unaltered levels of choline. The absence of gut microbiota also affects the immune system functions of the host, alters macrophage activity, and inhibits the IL-1β and IL-18 cytokine synthesis [44]. These findings suggest an impaired inflammatory response due to reduced inflammasome activation and the absence of caspase-1 activity, which confers protection against liver diseases [44].

**Figure 4:**
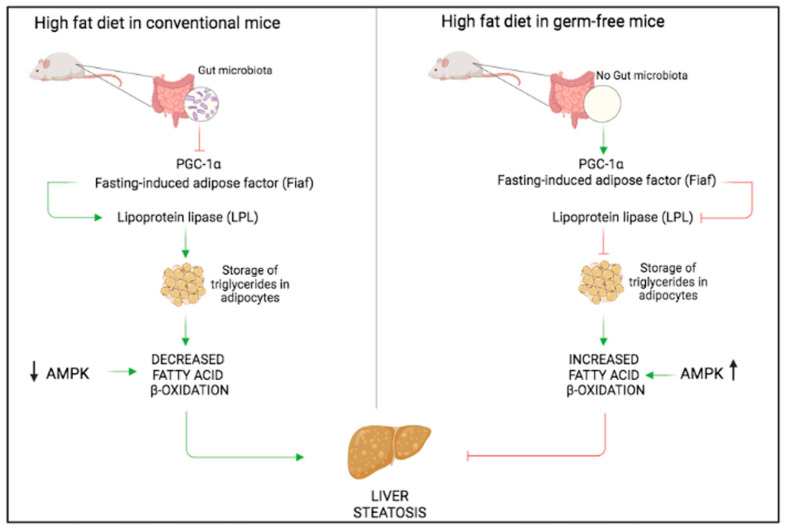
The gut microbiota regulates fat storage. The gut microbiota favors lipoprotein lipase (LPL) activation through inhibition of the fasting-induced adipose factor (Fiaf). This enhances triglycerides storage in adipose tissue and decreases fatty acid oxidation via suppression of AMP-activated protein kinase (AMPK) activity, thus leading to heightened adiposity and liver fibrosis. On the contrary, in germ-free mice, the absence of the gut microbiota favors resistance to diet-induced adiposity through inhibition of LPL and activation of AMPK.

Inflammation and cytokine release are one of the primary causes of insulin resistance associated with obesity. Insulin resistance compromises lipid uptake from the adipocytes and contributes to fatty acid systemic release [32]. AMP-activated protein kinase (AMPK) plays essential roles in mammalian energy homeostasis and metabolic process regulation. Therefore, AMPK finds implication in numerous metabolic disorders (diabetes, insulin resistance, obesity) [45]. Increased AMPK activity, as well as fatty acid oxidation, protect germ-free mice against diet-induced obesity [40]. However, an increased fatty acid oxidation may result from: (1) up-regulation of peroxisome proliferator-activated receptor-coactivator-1 (PGC-1α); (2) increased intestinal levels of fasting-induced adipose factor (Fiaf); (3) inhibition of lipoprotein lipase (LPL) and (4) altered cholesterol metabolism (Figure 4).

Germ-free mice, in fact, are characterized by the higher expression level of 3-hydroxy-3-methylglutaryl coenzyme A reductase gene (*HMGCR*)—involved in cholesterol biosynthesis—and up-regulation of membrane transporters for cholesterol excretion in the liver and small intestine [46]. This explains the reason why germ-free mice, after high-fat diet (HFD) consumption, display reduced levels of plasmatic cholesterol and increased levels of fecal cholesterol compared to their control counterparts.

In conclusion, these results demonstrate the intricate interplay between external factors (such as diet) and host factors (such as host genetics and gut microbiota) in predisposing or protecting against liver diseases and specifically NAFLD and NASH.

## 4. Colorectal Cancer and Microbiota

Colorectal cancer (CRC) is a common form of cancer: it is second in mortality and third in incidence [47]. As with the majority of diseases, CRC is multifactorial. Twin studies estimated the hereditability of CRC by about 12–35% [48], a result suggesting that the environment is the prevalent cause of CRC. Microorganisms cause about 15% of all cancers [49]: hepatitis B and C cause hepatocellular carcinoma; *Helicobacter pylori* gastric cancer; human papillomavirus cervical cancer. During the last ten years, in addition to the above-known microorganisms, the study included the gut microbiota. This community is essential for the physiology of the gastric tract, in particular for the correct functioning of the gastric immune system [50]. Changes in the abundance of single components alter the equilibrium leading to CRC and other forms of cancer [51]. An early study dated 1967 demonstrated that carcinogenic molecules need the presence of intestinal microorganisms to express their activity. The cycas (extract of *Cycas revoluta*) showed its carcinogenic property in conventional rats but not in germ-free rats. Repeated with the carcinogen 1-2 dimethyl hydrazine, the experiment yielded 93% of conventional rats—but only 21% of the germ-free rats—developed cancer [52]. Further studies demonstrated that specific intestinal microbiota species (*Escherichia*, *Enterococcus*, *Bacteroides*, and *Clostridium* genera) induce colorectal carcinogenesis. Further, the transfer of stools from patients with CRC causes intestinal cell proliferation in germ-free mice and tumor growth in conventional mice [53].

Human studies show that the gut microbiota from CRC patients differs from that of healthy individuals, displaying a lower abundance of protective taxa (*Roseburia*) and a higher abundance of carcinogenic taxa (*Bacteroides*, *Escherichia*, *Fusobacterium*, and *Porphyromonas*) compared to controls [54]. These results demonstrate the potential carcinogenic role of the microbiota.

Apart from bacteria, the human gut microbiota includes viruses and fungi. Molecular and histological tests have identified the presence of cytomegalovirus, John Cunningham virus [55], and human papilloma virus [55] in human CRC samples. In addition, a study that includes 74 patients with CRC and 92 healthy individuals identified 22 viral taxa that discriminate cases from controls [56]; an independent study reports the presence of bacteriophages [57], and fungi, respectively. In conclusion, the data accumulated during the last few years demonstrate that in the near future, microbiota may enter the arena of oncology.

## 5. Pathobionts

Microorganisms are the most represented species on our planet. Along with many other species, they colonize humans. The great majority of the microorganisms reside in our gastrointestinal tract. The microbiota provides functions essential to the host, such as the synthesis of vitamins, digestion of complex polysaccharides, preserves the intestinal epithelial barrier, and inhibits pathogen colonization [1]. Millions of years of co-evolution have irreversibly linked the health of mammals to their microbiota [58]. During this long co-evolution with the host, microbes have diverged, assuming multiple functions: many promote the health of the host (see above). However, some components of the gut microbiota cause diseases in the presence of environmental or genetic alterations in the host [59]. The components of the microbiota with pathogenic potential (pathobionts) induce chronic inflammation, while opportunistic pathogens cause acute inflammation; are innocuous to the host under normal conditions, while traditional pathogens may also cause disease in healthy hosts [60]. Pathobionts are also isolated from healthy individuals, can remain silent for decades before the disease becomes manifest, and can evolve while living in symbiosis with the host inducing inflammatory diseases [60].

The *Enterococcus gallinarum* (*E. gallinarum*) evolves into new strains that colonize the luminal or mucosal space in the gut [61]. *E. gallinarum* (EG) is a Gram-negative facultative anaerobe bacterium; it is detected in about 6% of human gut microbiota [62]. EG strains isolated from the liver and feces of autoimmunity-prone (NZW × BXSB) F1 mice were analyzed using the whole-genome sequencing (WGS) procedure [63]. The 11 isolates examined displayed 26 single-nucleotide variants and 10 small indels (insertions or deletions). A total of 13 genes exhibited non-synonymous mutations (a change in the DNA sequence coding for an amino acid different from the encoded one) or indels in at least two strains. The majority of these genes encode transcriptional regulators. Further, liver and fecal isolates were segregated into two site-specific lineages, suggesting the potential divergence of EG into two potential populations. In order to confirm this finding, groups of germ-free C57BL/6 mice were colonized with liver or fecal isolates. The EG isolated from the liver demonstrated a clear preference for liver translocation; the EG isolated from feces instead demonstrated a clear preference for fecal translocation. All the isolates displayed de novo mutations compared to the strain of origin, an indication that the original strain was replaced or significantly reduced in number.

## 6. Segmented Filamentous Bacteria (SFB)

Segmented filamentous bacteria (SFB) are Gram-positive *Clostridia* that adhere to Peyer’s patches in the mammalian small intestine, inducing IgA and B cell synthesis and activation of T-helper 17 (Th17) cells [64]. The production of Th17 cells and the IL-22 cytokine protect the host against enteric infection. Specific pathogen-free (SPF) mice colonized with SFB bacteria show greater numbers of Th17 cells in the gut and heightened protection against *Citrobacter rodentium* infection compared to mice without SFB [64]. Germ-free (GF) mice, which have very few Th17 cells in the gut, do not exhibit changes in Th17 level when reconstituted with a microbiota lacking SFB; rather, when reconstituted with SFB alone, show an increased number of intestinal Th17 cells [64]. However, the immunity conferred by SFB colonization may also come with a cost for the host. There is evidence that SFB may cause damage to the gut [65]. Mice with SCID (severe combined immunodeficiency)—reconstituted with CD4^+^CD45RBhigh T cells and colonized with SFB—develop severe colitis and intestinal inflammation [66]. In this particular animal model of colitis, SFB may synergize with the local microbiota and its immunomodulatory effect, which becomes excessive. Coherently with this hypothesis, mice mono-colonized with SFB do not develop intestinal pathology. Furthermore, the impact of SFB colonization on the host immune system of the host appears to extend beyond the gut, as SFB mono-colonization in GF mice increases the susceptibility of disease in animal models of rheumatoid arthritis and multiple sclerosis [60]. GF or antibiotic-treated animals display reduced Th17 cells outside the gut and did not develop the disease; this suggests that SFB alone can substitute for a complex microbiota in terms of driving pathology through Th17 cell induction [67]. This observation shows that gut bacteria can affect the extraintestinal health of the host. Collectively, these results illustrate that in the context of an autoimmune environment: (1) SFB alone, (2) the host alone (suffering from autoimmunity), or (3) both may promote disease.

## 7. Multiple Mutations

Recently it has emerged a great interest in somatic cancer mutations, which are present in 98% of the human non-coding genome. This great step ahead has been possible owing to advances in genomic technologies (including gene sequencing) and their reduced costs. Despite these advances, difficult challenges remain in understanding the potential relevance of somatic mutations identified in the non-coding human genome [68]. Many of these mutations display high frequencies, while few are rare. High-frequency mutations have uncertain biological significance. These results posed the problem of why mutations with apparently limited biological functions are the target of selection. The analysis of 60,954 cancer cases detected six cancer-specific oncogenes in which multiple mutations (MMs) occurred at high frequency. The role of MMs is still elusive [69]. At present, the prevalent hypothesis is that they confer fitness advantages to cancer cells, driving them to cancer [69]. The majority of the MMs are present in cis and show differential mutation patterns compared to single mutations, such as missense mutations versus in-frame indels. These properties suggest that MMs use their cis-acting effect as a mechanism to act as driver mutants, selecting the suboptimal mutations, which individually are functionally weak, but collectively account for a large proportion of oncogenic mutations [70]. In line with this suggestion, Ba/F3 cells transduced with major hotspot mutants of the PIK3CA (phosphatidylinositol 3-kinase) oncogene exhibited increased growth, while those transduced with minor hotspot mutants of the same gene did not display increased growth when compared with Ba/F3 wild-type-transduced cells. Notably, major and minor double mutants markedly enhanced proliferation compared with single mutants. These results suggest that individual suboptimal mutations can confer enhanced oncogenic potential to MMs. Since several types of cancer have the gut as a target, it is vital extending the research of somatic cis-regulator mutants to the types of cancers affecting the gut–liver axis.

## 8. An Attempt to Find a Rational to the Multiple Interactions between Gut Microbiota, Pathogens, and Host Interactions

The complexity of the gut microbiota originates from the need for both pathogens and microbiota to compete for nutrients. For instance, *Lactobacillus* species are unable to synthesize certain amino acids and must compete to take these essential molecules from the gut microbiota [71]. Mechanisms of competition have therefore evolved between pathogens and gut microbiota during their long co-evolution. Members of the microbiota produce bacteriocins and toxins that recognize and kill similar pathogens; this is the case of *E. coli*, which produces bacteriocins able to inhibit the enterohaemorrhagic *E. coli* (EHEC) [72]. Other components of the microbiota inhibit pathogen colonization altering the pH of the niche [73], thus enabling the innate immune system of the host to produce antimicrobial peptides [74]. Other components of the microbiota enforce the role of the intestinal epithelial barrier inducing it to release IgA that—binding to the microbial antigens—prevents infection [75]. Disruption of resident microbiota by antibiotics facilitates the overgrowth of pathogenic bacteria [76]. In addition, pathogens may use the microbiota to facilitate their colonization. By-products derived from the microbiota—such as bile salts—promote the germination of *Chlostridium difficile*, which causes diarrhea and colitis [77]. Viruses also can take advantage of gut microbiota. The mouse mammary tumor virus (MMTU) binds to the bacterial lipopolysaccharide to induce the production of the cytokine IL-10, which in turn depresses the antiviral immune response of the host, making the MMTU infection persistent.

Frequently is asked whether the microbiota is a friend or foe [78]. The context described above makes the answer intricate. At present, it is clear that the microbiota has a large importance for the host (see above). However, we do not adequately know either the host or the microbe properties. The concept of microbial virulence does not explain the emerging and rapid diffusion of infectious diseases caused by microbes—such as *Candida albicans*—for a long time classified as non-virulent. In 1950, the use of antibiotics gave origin to a high number of oral candidiasis cases [79]. Patients with AIDS are more susceptible to pneumococcal pneumonia. Thus, neither a microbe nor a host alone can explain how the same microbe (*Streptococcus pneumonia*), clearly virulent, may behave as a pathogen in one host and as opportunistic in another host.

In conclusion, the damage originates from (1) a microbe (including the microbiota; (2) the host, or (3) both. In other words, there are only microbes and hosts that interact, with the result being the outcome of their interactions.

## 9. Conclusions

There is conclusive evidence that the alteration of gut microbiota causes multiple liver diseases [80,81,82,83]. At the same time, several treatments (prebiotics, probiotics, and fecal transplantation) have given encouraging results in the treatment of NAFLD. Further, animal models mimicking a human disease are helping to better understand many important pathways and extend these results to humans [84]. Patients with alcoholic hepatitis who received one week of fecal microbiota transplantation (FMT) from healthy donors showed improved liver function and survival [41]. Moreover, patients with cirrhosis, which received FMT from accurately selected donors, needed shorter therapy and exhibited improved cognitive tests, increased microbial diversity, and beneficial taxa [85]. In addition, several clinical trials are scheduled to investigate the effect of FMT on NASH, chronic hepatitis B, obesity, and type 2 diabetes [84]. These results suggested that therapies limiting the growth of harmful bacteria (such as new generations of probiotics, bacterial metabolites, antimicrobial peptides, fecal microbial transplantation, and phages that target specific bacteria) represent new potential therapeutic approaches. However, at present, these therapies lack specificity for the target disease. In addition, their repercussions on liver cancer cells are still not clear, and differences in intestinal bacteria between different liver diseases and different individuals have been detected. Whether these differences might help to discriminate between liver diseases or patients’ stratification, further research is needed. In conclusion, the above results encourage thinking that not far in the future, several liver diseases will be resolved with personalized medicine grounded on the exploitation of the patient’s gut microbiota. At the same time, to understand the adverse effects that follow the alteration of gut microbiota, additional studies are required. However, these effects cannot be detected using conventional biochemical and histopathological investigations but require high-through “omics” analyses.

Once delineated what at present is known about the gut microbiota-host metabolism interaction, it is interesting mentioning the therapeutic possibilities that might emerge from a better understanding of gut microbiota. Numerous studies have demonstrated that gut microbiota influences insulin resistance, dyslipidemia, atherosclerosis, hepatic steatosis, and elevated blood pressure [86]. The next step will be to understand how gut microbiota influences the above diseases; it will require monitoring microbial changes over time, genetic and epigenetic effects on the immune system, and diet changes; these data will be crucial for a personalized manipulation of gut microbiota [87]. Metabolic diseases generally are attributed to the translocation of endotoxin (LPS) of Gram-negative microbes, which cause low-grade inflammation. However, this conclusion is based on uncertain data. This topic is second to be better understood; its corrected knowledge might lead to more appropriate therapies for several diseases [88].

Until recently, the majority of intestinal microbes were thought of as not culturable. However, using the growth medium YCFA [89], most of the anaerobic microbiota were cultured with success [89]. Of the 137 distinct species isolated, 90—listed on the Human Microbiome Project as “most wanted”—were classified as uncultured microbes [89]. These results will hopefully lead to further insights into the function and interactions between various gut microbes and help to understand which cases will respond to FMT. At present, this issue remains a vital question. Moreover, it is not only the question of which microbes should be infused, but also how many species or strains are needed to alter the gut microbiota effectively [90]. Studies to date have mainly been limited to genus- and species-comparisons and have not clarified to which extent donor microbiota colonizes a recipient [91]. Recently it has been observed that effective colonization by donor fecal bacteria is influenced by the gut microbial composition of the recipient and differs between metabolic diseases [91].

## Data Availability

Not applicable.
